# Trivial Neck Bite Resulting in a Stroke in an 18-Month-Old Child: A Case Report

**DOI:** 10.7759/cureus.56544

**Published:** 2024-03-20

**Authors:** Ruthwik Duvuru, Ahmad Alawadhi, Shivani Raju, Haitham ElBashir

**Affiliations:** 1 College of Medicine, Mohammed Bin Rashid University of Medicine and Health Sciences, Dubai, ARE; 2 College of Medicine, The Oxford Medical College, Hospital & Research Centre, Bangalore, IND; 3 Pediatric Neurology, Al Jalila Children's Speciality Hospital, Dubai, ARE

**Keywords:** clinical neuroscience, pediatric stroke, inpatient pediatrics, pediatric seizure, vascular neurology

## Abstract

Stroke is often viewed as a diagnosis found In the elderly with or without comorbidities, but it is vital to not rule it out in a pediatric patient presenting with signs and symptoms of stroke. Here, we present a case of an 18-month-old boy who arrived at the emergency department with left arm weakness and left-sided seizures a few minutes after a right-sided trivial neck bite that was initially overlooked by the parents until symptoms occurred. Urgent imaging further with a computed tomography scan of the brain revealed a hypodense lesion in the area covering the lateral part of the frontal lobe, insula, and parietal cortex of the right hemisphere. Subsequent CT cervical-cerebral angiogram revealed normal aortic arch, carotid, and vertebral arteries with no dissection, stenosis, or occlusion. However, there was a 4 mm-long occlusion of the M2 segment of the right middle cerebral artery (MCA) suggestive of emboli and subsequent low attenuation of the brain parenchyma in the anterior aspect of the right MCA vascular territory corresponding with the infarction. The objective of this case report is to educate and inform both parents and medical professionals regarding the risk of neurological damage that can occur with minor head and neck trauma that is often overlooked and therefore the importance of ruling it out with necessary imaging modalities.

## Introduction

Stroke is among the top 10 causes of mortality in infants and adolescents and is a neurological injury caused either due to an occlusion or rupture of a blood vessel. The World Health Organization describes stroke as a clinical syndrome of focal or global disturbance of brain function over 24 hours or leading to death with no obvious nonvascular cause and about 50% of children presenting with focal neurological deficits have at least one risk factor that was previously identified. Sickle cell disease, heart disease, chronic anemia, head trauma, and dehydration are some of the risk factors associated with stroke [1,2].

Although stroke is often viewed as an incident in the elderly, it also occurs in the pediatric age group, albeit rarer. The annual incidence rate of acute ischemic stroke (AIS) in infants ranges from 0.6 to 7.9 per 100,000 children per year and has been reported to exponentially increase with age [3-5]. Tsze and Valente reported in their 2011 review that the most common clinical characteristic of stroke was hemiparesis or focal central nervous system (CNS) deficit, followed by altered mental status, headache, and seizure [6]. Signs and symptoms of stroke can vary from lethargy, apnea spells, or hypotonia in infants to protean symptoms, such as deterioration in general condition, increased crying in toddlers, and specific neurological deficits in older children. Other disease-mimicking strokes include complicated migraines, intracranial neoplasms, and focal seizures with Todd's paralysis.

According to reports, children who sustain blunt trauma have a 0.03-1.3% incidence of blunt cerebrovascular damage, whereas pediatric traumatic brain injury (TBI) has a 3.5% incidence. Accidents involving motor vehicles are the most common cause of injury. However, it is noteworthy that penetrating cerebrovascular damage in pediatric trauma is a problematic condition in and of itself while being infrequent. It has been observed that 5.4% of pediatric patients who underwent particular screening for penetrating trauma, including gunshot wounds, had penetrating cerebrovascular damage [2].

Suspected stroke cases are diagnosed via non-contrast head computed tomography (CT) as it is sensitive to acute bleeding; however, magnetic resonance arteriography (MRA) and magnetic resonance venography should also be carried out to note vessel patency and vascular anatomy [6].

Although there are no established laboratory testing guidelines for the assessment of pediatric stroke, several studies suggest liver function tests, C-reactive protein, erythrocyte sedimentation rate, brain MRI, MRA, and MRV CT angiogram [6].

Due to the likelihood of children having an underlying condition that would benefit them for anticoagulation, recent guidelines suggest short-term anticoagulation pending evaluation of stroke etiology. However, thrombolytic therapy in children with ischemic strokes should done carefully as there is not sufficient literature apart from a few case reports discussing the application of tissue plasminogen activator (tPA). Long-term anticoagulation could be provided in the form of aspirin, clopidogrel, and warfarin [1,7,8].

Herein, we report a case of an otherwise healthy child who developed a stroke following trivial neck trauma at the age of 18 months.

## Case presentation

An 18-month-old male child who was previously healthy presented to the emergency department at another private hospital with left arm weakness and left-sided seizures a few minutes after a right-sided trivial neck bite by a young fellow party attendant. The site of the bite was visibly bruised and erythematous to the naked eye but had no obvious bleed. The seizures were focal and clonic, with the left upper extremity shaking and stiffening. They were brief, occurring for a few seconds, but recurrent. He was started on levetiracetam 160 mg BID, and later IV phenytoin was added to control his seizures. However, the patient was vitally stable (Table 1), and an urgent CT scan of the brain revealed a hypodense lesion in the area covering the lateral part of the frontal lobe, insula, and parietal cortex of the right hemisphere.

**Table 1:** Vitals on the initial presentation to the emergency department

Vitals	
Temperature (tympanic)	36.0 degrees Celsius
Blood pressure	100/59
Pulse	130
Respiratory rate	24
Spo_2_	99%

**Figure 1:**
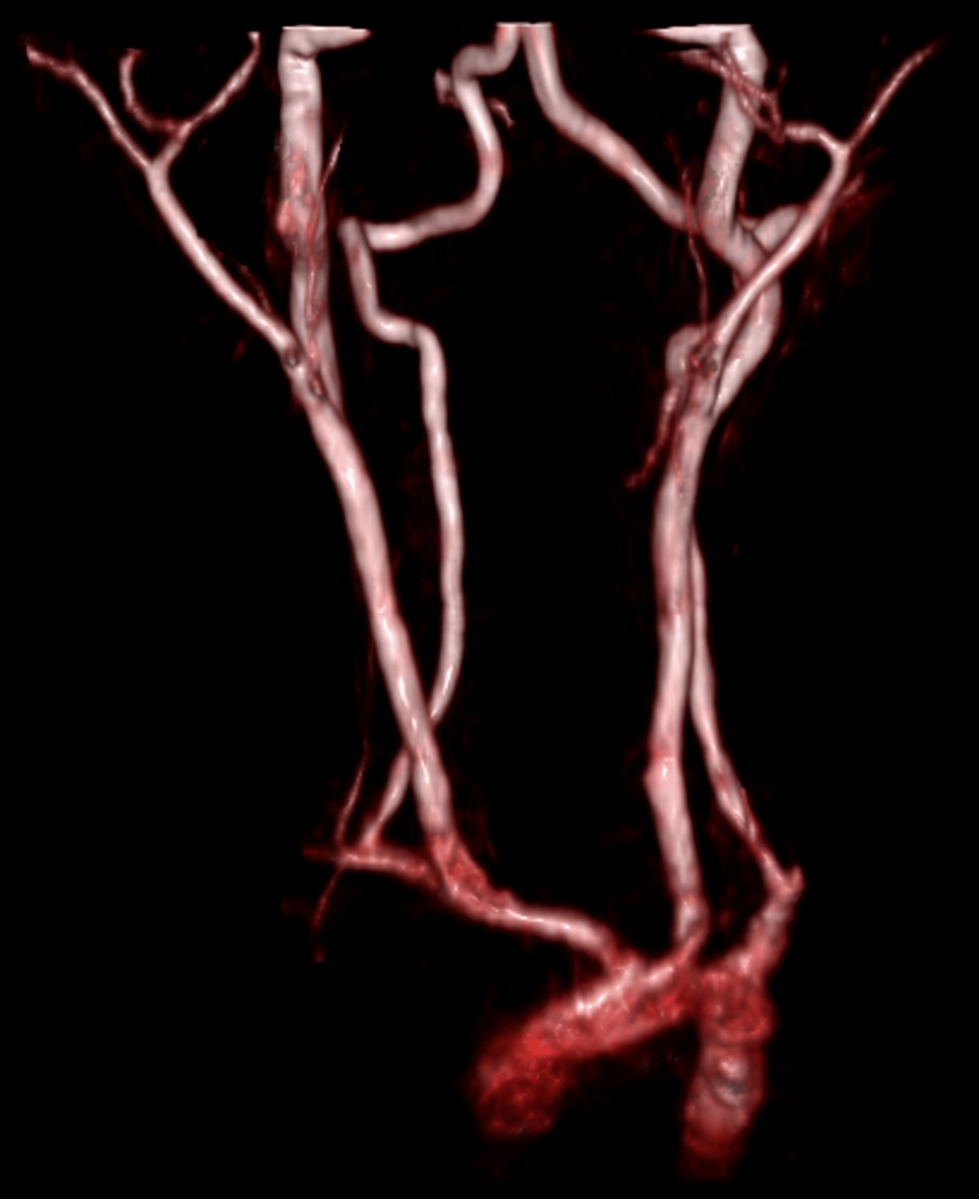
MRA of the carotids and cerebral arteries, visualized portion of the aortic arch, both common carotid, internal carotid and vertebral arteries in the neck appear unremarkable. Normal caliber of the arteries. No evidence of dissection. MRA: magnetic resonance angiography

Subsequent CT cervico-cerebral angiogram (Figure 1) revealed normal aortic arch, carotid, and vertebral arteries with no dissection, stenosis, or occlusion. However, there was a 4 mm-long occlusion of the M2 segment of the right middle cerebral artery (MCA) suggestive of emboli and subsequent low attenuation of the brain parenchyma in the anterior aspect of the right MCA vascular territory corresponding with the infarction. A cardiac echocardiogram was reported as normal. Further tests revealed normal thrombophilia screening, but the child had iron deficiency anemia. Before the incident, the patient had normal development with age-appropriate milestones and was up to date with vaccinations. The patient's medical history was insignificant for any previous history of stroke, perinatal complications, or allergies to food or drugs.

**Figure 2:**
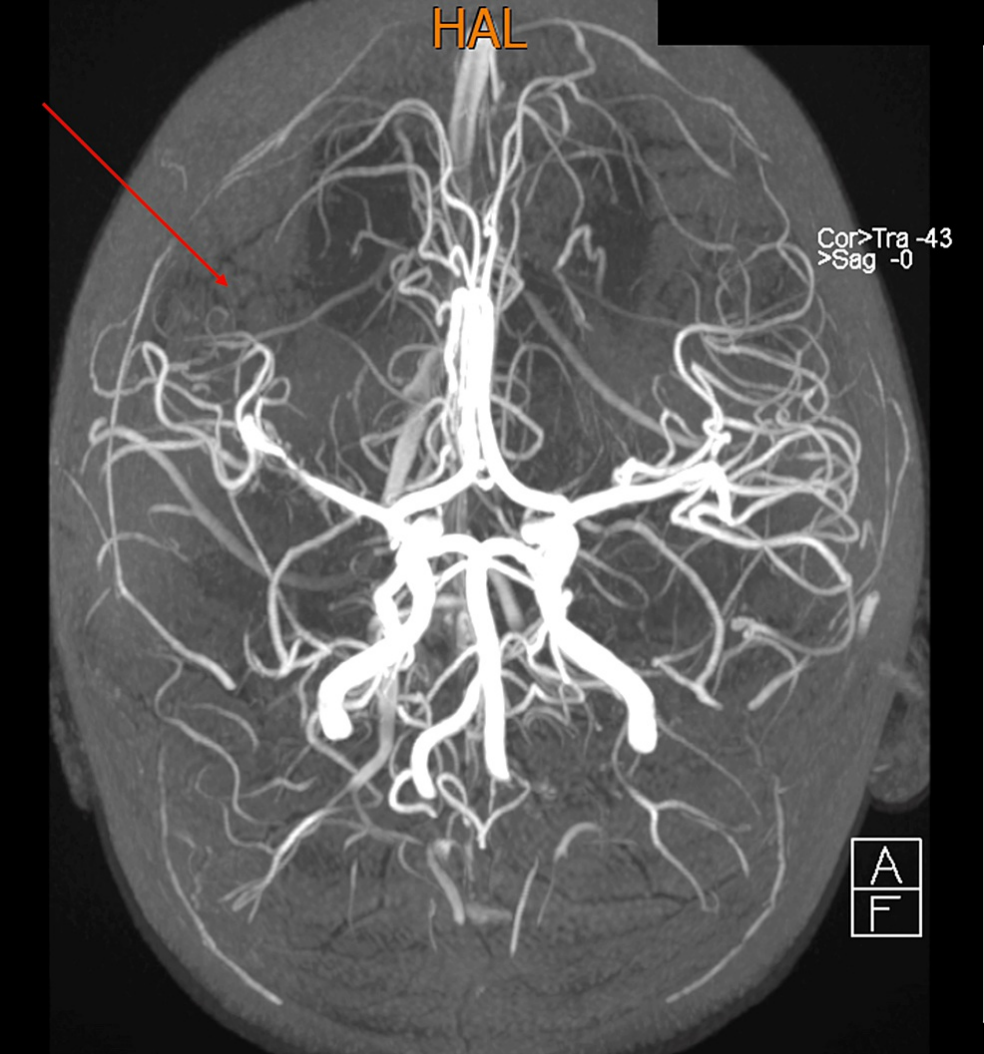
MRA of the brain revealing tight stenosis of the M1 segment of the right middle cerebral artery with paucity of the distal branches of the middle cerebral artery is seen on the right. MRA: magnetic resonance angiography

**Figure 3:**
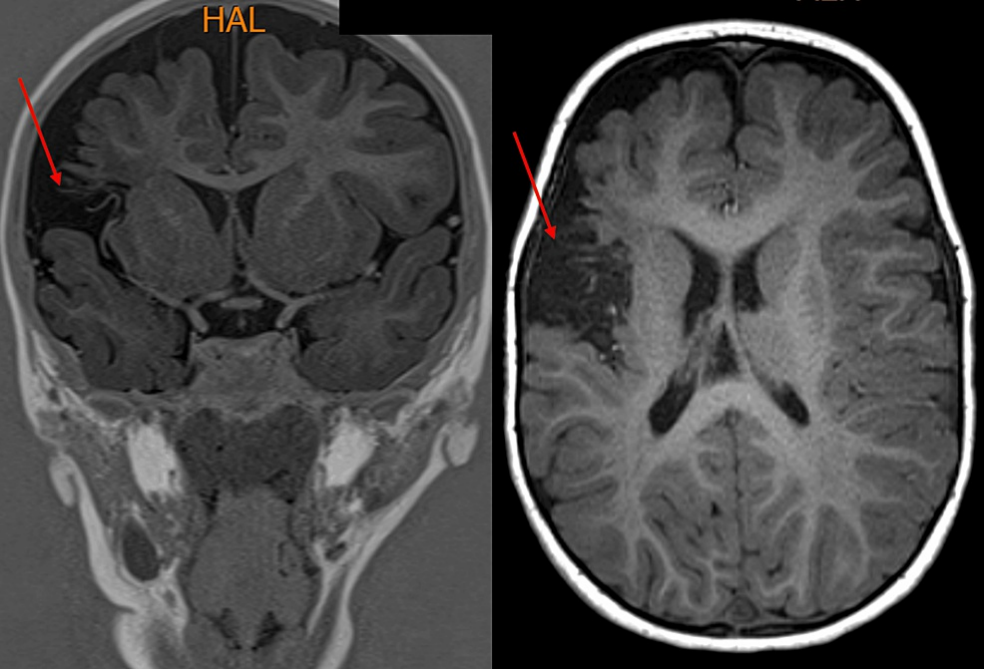
There is volume loss in the right frontal lobe, mainly around the insular region. There are cortical loss and some abnormal signals in the white matter representing gliosis. This represents encephalomalacia in the right middle cerebral artery territory.

A follow-up clinical visit at our center revealed a normal cranial nerve examination, but on motor examination of the left side, he had increased tone and brisk reflexes on both upper and lower limbs with decreased strength and left wrist drop. He had an extensor plantar response. He had a mild left-sided hemiparetic gait and decreased left-arm swing. The rest of his examination was normal, including a normal neurological examination of the right side. The patient was subsequently referred to the physiotherapy department. He improved with physiotherapy but continued to have left-sided hemiparesis. The brain MRI scans (Figures 2, 3) done a month later at our center were both consistent with the initial CT scans.

## Discussion

Although post-stroke seizure and post-stroke epilepsy are common causes of hospital admission, after a stroke, the relation between both is quite complex [9]. Shinton et al. reported that in patients with acute stroke, seizures might be a precursor of stroke. Another study reports that the risk of stroke in patients with preexisting seizures is almost threefold when compared to controls [10]. Sundelin et al. in their nationwide cohort study of 1,220 children under the age of 18 reported that the risk of epilepsy after AIS is increased and was the highest within the first six months and remained elevated even 20 years later [11]. Although post-stroke seizure management is beyond the scope of this report, physicians taking care of children with stroke should have high suspicion for clinical or subclinical seizures in an acute setting. Fox et al. have reported that children with acute seizures at the time of stroke represent a particularly vulnerable population with four times the risk of remote seizures and a fourth of these patients eventually develop epilepsy [12].

The common findings in pediatric patients with stroke post-neck trauma would be arterial dissection or the presence of a thrombus on the MRA of the neck or brain. However, in our patient, the MRA scans reported no such findings. A study by Munot et al. conducted a retrospective review of clinical records and imaging of pediatric patients with AIS and normal MRA to compare it with children with AIS and abnormal MRA and thus reported that the former group of patients are not distinct but are more likely to have single territory lesions and have nonvascular risk factors [13]. However, our patient did not have any risk factors at the time of stroke, which adds to the atypical nature of our case.

Finally, early recognition of stroke and long-term multidisciplinary management is important, and current strategies for acute management of pediatric stroke remain the same as that of an adult. The Thrombolysis in Pediatric Stroke (TIPS) study tried to answer two concerns: the safety of tPA in pediatric stroke patients and the outcomes of tPA use in childhood stroke. The study was closed due to a lack of sufficient patient enrollment, but it established systems for the evaluations and management of stroke. Based on the TIPS study, the American Stroke Association (ASA) reported that the adult dose of 0.9 mg/kg of IV tPA would be conservative in pediatrics considering the developmental differences in plasminogen levels [14].

## Conclusions

Stroke can cause severe morbidity and death in children, even though it is a very uncommon occurrence. Optimizing outcomes for children with strokes will need an understanding of how they present, as children with strokes frequently have distinct risk factors compared to adults. Even though juvenile stroke occurrences are on the rise, detection is frequently delayed, and patients may continue to get an incorrect or inadequate diagnosis.
